# Supplementary material for: Effects of the Entomopathogenic Fungus Mucor hiemalis BO-1 on the Physical Functions and Transcriptional Signatures of Bradysia odoriphaga Larvae
Source: Insects. 2023 Feb 8;14(2):162. doi: 10.3390/insects14020162 (PMC9964685; doi:10.3390/insects14020162)
Supplement: Supplementary file 1 [file insects-14-00162-s001.zip › Table S1.pdf]

Table S1. Quality of the RNA isolated from the biological samples used for transcriptome analysis

Sample names	Treatments	RNA concentrations (ng/μl)	OD value (260/280)	OD value (260/230)
Normal-1	Healthy larva	807.3	2.18	2.49
Normal-2	Healthy larva	790.9	2.22	2.53
Treat-1	Diseased larva	783.9	2.18	2.43
Treat-2	Diseased larva	713.2	2.18	2.51
