# Supplementary material for: Effects of the Entomopathogenic Fungus Mucor hiemalis BO-1 on the Physical Functions and Transcriptional Signatures of Bradysia odoriphaga Larvae
Source: Insects. 2023 Feb 8;14(2):162. doi: 10.3390/insects14020162 (PMC9964685; doi:10.3390/insects14020162)
Supplement: Supplementary file 1 [file insects-14-00162-s001.zip › Table S2.pdf]

Table S2. Primers used in reverse transcriptase-quantitative polymerase chain reaction (RT-qPCR)

Gene	Gene ID	Primer sequence (5′→3′)	Fragment length (bp)	GenBank accession
uidA	HA402_000028	5′-TAACGCGAACCCATGCTTCC-3′<br>5′-TTGGGCACTCCACGAATAAG-3′	215	OP893620
PRSS	HA402_001933	5′-ACAACACTTATGGTTCCCAAC-3′<br>5′-CCAGAATCACCCTGACATGC-3′	166	OP893630
amyA	HA402_001429	5′-CTCCTGCCGATCCTACAACC-3′<br>5′-TACCGCGACAAAACGCAATC-3′	195	OP893621
LIPA	HA402_001695	5′-AGCGTCAGAGTTGTTCACAG-3′<br>5′-CTGCAAATGATCAACCAGCG-3′	216	OP893622
treA	HA402_003401	5′-ACTGGTGACACGGATTTTCTC-3′<br>5′-CTCTTTTTCGATGTCGGTTGC-3′	198	OP893623
CPT1A	HA402_014097	5′-ACCTATCATGGCTCAGGGAC-3′<br>5′-GACCGTGTTGCTCAATAGTC-3′	176	OP893624
PXDN	HA402_012831	5′-AGCACAGTTATCGGGCTGTC-3′<br>5′-TGTCCATCCAATCGGTGAGC-3′	196	OP893625
catB	HA402_001366	5′-CCCATTTCGATCGTGAACGC-3′<br>5′-ATCACGAGCGGTATCTGCTG-3′	203	OP893626
gst	HA402_005674	5′-GAGATTCCAGTGCTTGACGAC-3′<br>5′-TACAATAGCCCGCTTGACGG-3′	169	OP893627
SOD1	HA402_011746	5′-AGTTGCTTACCTTACTGGCG-3′<br>5′-TGTCTGGATTGAAGTGAGCG-3′	188	OP893628
CYP9	HA402_008470	5′-ATGGTATTGCTACAGCTGCC-3′<br>5′-ATAGAGCTTCGGTGCAATGG-3′	155	OP893629
ECSIT	HA402_001791	5′-ACTAAATACGTTCGGGCGTC-3′<br>5′-ATCAACCGTACACATTCGGG-3′	166	OP893631
